# Supplementary material for: Bayesian refinement of protein structures and ensembles against SAXS data using molecular dynamics
Source: PLoS Comput Biol. 2017 Oct 18;13(10):e1005800. doi: 10.1371/journal.pcbi.1005800 (PMC5662244; doi:10.1371/journal.pcbi.1005800)
Supplement: S1 Archive — (BZ2) [file pcbi.1005800.s011.tar.bz2 › gromacs_4.62_bayesian_saxs_refi/share/html/online/g_rmsdist.html]

g\_rmsdist


| g\_rmsdist Main Table of Contents |

**VERSION 4.6  
Sat 19 Jan 2013**

---

### Description

g\_rmsdist computes the root mean square deviation of atom distances,
which has the advantage that no fit is needed like in standard RMS
deviation as computed by g\_rms.
The reference structure is taken from the structure file.
The RMSD at time t is calculated as the RMS
of the differences in distance between atom-pairs in the reference
structure and the structure at time t.

g\_rmsdist can also produce matrices of the rms distances, rms distances
scaled with the mean distance and the mean distances and matrices with
NMR averaged distances (1/r^3 and 1/r^6 averaging). Finally, lists
of atom pairs with 1/r^3 and 1/r^6 averaged distance below the
maximum distance (-max, which will default to 0.6 in this case)
can be generated, by default averaging over equivalent hydrogens
(all triplets of hydrogens named \*[123]). Additionally a list of
equivalent atoms can be supplied (-equiv), each line containing
a set of equivalent atoms specified as residue number and name and
atom name; e.g.:

3 SER HB1 3 SER HB2

Residue and atom names must exactly match those in the structure
file, including case. Specifying non-sequential atoms is undefined.

### Files

| option | filename | type | description |
| --- | --- | --- | --- |
| **-f** | traj.xtc | Input | Trajectory: xtc trr trj gro g96 pdb cpt |
| **-s** | topol.tpr | Input | Structure+mass(db): tpr tpb tpa gro g96 pdb |
| **-n** | index.ndx | Input, Opt. | Index file |
| **-equiv** | equiv.dat | Input, Opt. | Generic data file |
| **-o** | distrmsd.xvg | Output | xvgr/xmgr file |
| **-rms** | rmsdist.xpm | Output, Opt. | X PixMap compatible matrix file |
| **-scl** | rmsscale.xpm | Output, Opt. | X PixMap compatible matrix file |
| **-mean** | rmsmean.xpm | Output, Opt. | X PixMap compatible matrix file |
| **-nmr3** | nmr3.xpm | Output, Opt. | X PixMap compatible matrix file |
| **-nmr6** | nmr6.xpm | Output, Opt. | X PixMap compatible matrix file |
| **-noe** | noe.dat | Output, Opt. | Generic data file |

### Other options

| option | type | default | description |
| --- | --- | --- | --- |
| **-[no]h** | bool | no | Print help info and quit || **-[no]version** | bool | no | Print version info and quit || **-nice** | int | 19 | Set the nicelevel || **-b** | time | 0 | First frame (ps) to read from trajectory || **-e** | time | 0 | Last frame (ps) to read from trajectory || **-dt** | time | 0 | Only use frame when t MOD dt = first time (ps) || **-[no]w** | bool | no | View output .xvg, .xpm, .eps and .pdb files || **-xvg** | enum | xmgrace | xvg plot formatting: xmgrace, xmgr or none || **-nlevels** | int | 40 | Discretize RMS in this number of levels || **-max** | real | -1 | Maximum level in matrices || **-[no]sumh** | bool | yes | Average distance over equivalent hydrogens || **-[no]pbc** | bool | yes | Use periodic boundary conditions when computing distances |

---

http://www.gromacs.org  
gromacs@gromacs.org
